# Thermodynamics, kinetics and isothermal studies of tartrazine adsorption onto microcline/MWCNTs nanocomposite and the regeneration potentials

**DOI:** 10.1038/s41598-023-37181-2

**Published:** 2023-06-19

**Authors:** James Friday Amaku, Raymond Taziwa

**Affiliations:** Department of Applied Science, Faculty of Science Engineering and Technology, Walter Sisulu University, Old King William Town Road, Potsdam Site, East London, 5200 South Africa

**Keywords:** Environmental sciences, Chemistry, Materials science, Nanoscience and technology

## Abstract

The quest for a cheap, effective, and eco-friendly wastewater treatment technique that is free of secondary toxic byproducts, calls for the fabrication of a nature-friendly adsorbent with a robust capacity to decontaminate polluted water sources and be recycled. To this end, we report the fabrication of novel nanocomposite (KMCM) from microcline (KMC) and multiwall carbon nanotubes (MWCNTs). The adsorbents (KMC and KMCM) were characterized using XRD, BET, SEM, TGA and FTIR. The novel and low-cost nano sorbent were designed for the elimination of tartrazine (Tatz) from wastewater. The adsorption of Tatz onto KMC and KMCM was influenced by adsorbent dose, initial Tatz concentration, contact time and solution pH. Experimental data acquired from the equilibrium studies were well addressed by the Langmuir isotherm model. The maximum uptake capacity of 37.96 mg g^−1^ and 67.17 mg g^−1^ were estimated for KMC and KMCM. The kinetics for the adsorption of Tatz onto KMC and KMCM was best expressed by pseudo-second-order and Elovich models. The thermodynamic parameters revealed that the uptake of Tatz onto KMC and KMCM was an endothermic (ΔH: KMC = 35.0 kJ mol^−1^ and KMCM = 42.91 kJ mol^−1^), entropy-driven (ΔS: KMC = 177.6 J K^−1^ mol^−1^ and KMCM = 214.2 J K^−1^ mol^−1^) and spontaneous process. Meanwhile, KMCM demonstrated good reusability potential and superior adsorption efficiency when compared to other adsorbents.

## Introduction

The increasing world population, exponential growth of the modern manufacturing industry and advances in industrial technology are cardinal contributing factors responsible for water pollution^1^. Many inorganic and organic toxic materials have been reported to adversely affect the physicochemical properties of aquatic ecosystems amongst which are dyes^2^. Among the inorganic pollutants are dyes. Dyes are organic compounds used by several industries to impact colour and are classified as anionic, cationic, and nonionic. Meanwhile, industries such as pharmaceutical, paper, paint, textile, and food amongst others are known as principal consumers of dyes^3^. Tartrazine is an anionic yellow dye, consisting of sulphonic, azo (N=N) and carboxylic functional groups and is frequently employed as an additive in consumables such as sweet ice cream, beverages, gelatins, chips, chewing gum, bread, yoghurt and pharmaceutical^4^.

Besides the useful benefit of tartrazine, reports revealed that tartrazine has the potential to cause hypersensitivity, allergy, skin eczema, asthma, mutation, cancer, and immunosuppressive effects^5^. In a bid to sequester water contaminants from the aquatic ecosystem, different types of physicochemical/biological methods have been employed. Some of these treatment techniques include electrochemical techniques^6^, biological treatments^7^, extraction^8^, ion exchange^9^, filtration^10^, photodegradation^11–14^, chemical precipitation^15^, membrane bioreactor^16^ and reverse osmosis^17^. On the other hand, the application of these methods for pollutant removal is limited and this is due to the possible generation of a toxic secondary pollutant, elevated operational cost and inefficiency at low pollutant concentration^18,19^. Considering the adverse implication of the aforementioned challenges, it is, therefore, necessary to design an eco-friendly and cost-effective technique for water purification.

Adsorption has been reported to be efficient for the eradication of dye even at low concentrations with low running cost of operation, excellent selectivity and ease of operation^20^. Extensive study has been done using adsorbents of both inorganic and organic origin for the sequestration of dyes. Amongst these adsorbents include; cellulose^21^, biogas waste^22,23^, montmorillonite^24^, waste peel^25^, aerogels^26,27^, nanocomposite^28,29^, Zn/Al-LDH^30^, banana pith^31^, coconut mesocarp^32^, peat^33^ graphene oxide^34^, chitin^35^, iron oxide nanoparticles^36^, chitosan^37^, silica^38^, jute stick powder^39^, peanut hull^40^, polypyrrole/SrFe_12_O_19_/graphene^41^, jute processing wastes^42^, activated carbon^43–45^, soy meal hull^46^, quartz waste^47^, rice husks^48^, maize stalks^49^ Fe/zeolite^50^, hazelnut shells^51^, seeds^52^, husk^53^, Scots pine^54^, kaolinite clay^55^ and leaves^56^. However, some of the adsorbents previously mentioned were found to have some degree of demerit such as poor filtering, ineffective at high temperatures, expensive to regenerate and limited selectivity. Hence, it is important to design an adsorbent with exceptional quality for the removal of Tatz from wastewater. Microcline (KAlSi_3_O_8_) is composed of potassium, aluminium and silicate, it is generally known as potassium feldspar and crystallizes in the triclinic system^57^. This mineral can be obtained as igneous, sedimentary or metamorphic rock. As a mineral, microcline is ubiquitous and can be modified as an adsorbent for water remediation practices. On the other hand, carbon nanotubes have demonstrated exceptional physical and chemical properties, and these characteristics have resulted in the successful application of CNTs in different fields^58,59^. Meanwhile, CNTs have demonstrated excellent capacity to sequester both organic and inorganic pollutants from the aqueous phase^60,61^.

Hence, the application of MWCNTs as a modifier for microcline (KMC) was performed to obtain a nanocomposite (KMCM) with a superior capacity for tartrazine adsorption. This study further assessed cardinal adsorptive factors, such as contact time, adsorbate pH, the effect of dosage, initial Tatz concentration and solution temperature. As far as we are aware, the application of functionalized MWCNTs as microcline modifier and their use as wastewater treatment agents has not been reported.

## Experimental

### Material and chemicals

Tartrazine (Tatz), multi-walled carbon nanotubes (MWCNTs), sulfuric acid (H_2_SO_4_), glutaraldehyde, sodium chloride (NaCl), nitric acid (HNO_3_), hydrochloric acid (HCl), ethanol (C_2_H_6_O), and sodium hydroxide (NaOH) were acquired from Sigma-Aldrich.

### Sample preparation

The microcline rock sample was kindly furnished by the Department of Geology, Michael Okpara University of Agriculture Umudike, Nigeria. The stony sample was thereafter processed in a ball mill to obtain a fine powder. The milled microcline was then washed with deionized water under vacuum, oven-dried, labelled (KMC), and stored in an airtight container for future application.

### Preparation of nanocomposite

2 g of KMC and 0.5 g of MWCNTs were weighed into a beaker containing 100 cm^3^ of 6 mol dm^−3^ HCl, the mixture was then stirred for 3 h, diluted, filtered, and washed to neutral under vacuum. The product obtained was then dried and treated with 100 cm^3^ of 6 mol dm^−3^ HNO_3_ for 3 h. Thereafter, 2 g of the purified composite was then weighed into a 250 cm^3^ beaker containing 125 cm^3^ of an acid mixture (nitric acid-sulfuric acid (3:1 (V/V)), stirred for 6 h, diluted, filtered, and washed to neutral under vacuum. The product obtained is then resuspended into 50 cm^3^ of deionized water containing 2 cm^3^ of 2% glutaraldehyde. The mixture is stirred for 1 h, filtered, vacuum oven-dried, labelled (KMCM), and stored in an airtight container for future use.

### Characterization

The microstructure and surface morphology of KMC and KMCM were assessed using field emission scanning electron microscopy (FESEM) (ZEISS ultra plus). The XRD patterns of KMC and KMCM were validated using an X-ray diffractometer (XRD Bruker D8 Advance powder X-ray diffraction). The behaviour of KMC and KMCM at elevated temperatures was examined via a simultaneous thermal analyzer (Mettler Toledo TGA/DSC1 Star System). The chemical status of pristine (KMC and KMCM) and tartrazine-loaded adsorbent (KMC-Tatz and KMCM-Tatz) adsorbents were assessed using Fourier transform infrared (FTIR) spectroscopy (Thermo Nicolet-870 spectrophotometer). The pore volume, pore diameter and surface area of KMC and KMCM were obtained using the nitrogen sorption–desorption methods by Barrett-Joyner-Halenda (BJH) and Brunauer–Emmett–Teller (BET) (Micromeritics Instruments Corp., USA).

### Batch adsorption

The capacity of KMC and KMCM to remove Tatz from the aqueous solution was investigated using the batch adsorption technique. 1 g of Tatz powder was weighed into 1 dm^3^ of deionized water to prepare the stock solution (1000 mg dm^−3^). The working solution (100 mg dm^−3^) was prepared from the stock solution via serial dilution and adjusted to desired solution pH using 0.1 mol dm^−3^ NaOH, or 0.1 mol dm^−3^ HCl solutions. The batch adsorption of Tatz was achieved by contacting KMC or KMCM (0.05 g, 150 rpm) with Tatz solution (100 mg dm^−3^, 25 cm^3^) in a 100 cm^3^ stoppered glass bottle for 1440 min at room temperature. The mixture was filtered, and the equilibrium concentration of Tatz was determined using ultraviolet–visible (UV–vis) spectrophotometry (λ_max_ = 426 nm)^62^. The implication of dosage, contact time, solution temperature, solution pH and initial adsorbate concentration were assessed for the optimization of the removal process. Meanwhile, the uptake capacity (see Eq. S1), removal efficiency (see Eq. S2)^63^, kinetics (see Table S1), and isotherm (see Table S2) analysis of Tatz adsorption onto KMC and KMCM were determined as described in the supplementary information S1.

### Reusability

To demonstrate the reusability of the KMC and KMCM for the removal of Tatz, aqueous ethanol was used as the eluting agent. Briefly, 0.5 g of KMC or KMCM was contacted with 250 cm^3^ of 100 mg dm^−3^ Tatz solution and shaken for 1440 min at room temperature to adsorb Tatz. The mixture was filtered, washed, and dried. 0.05 g of the spent adsorbents (KMC-Tatz or KMCM-Tatz) were then eluted using 25 cm^3^ of aqueous ethanol for 30 min at room temperature. The adsorption efficiency of KMC and KMCM was calculated using Eq. (S2) describes in the supplementary information S1.

### Determination of pH point of zero charge

The pH at the point of zero charge (pH_PZC_) of KMC and KMCM is a vital factor in unfolding the ionization behaviour of these novel adsorbents. The pH_PZC_ of KMC and KMCM was achieved using the previously described method^64^. 50 cm^3^ of 0.01 M NaCl was measured into eleven conical flasks and adjusted to pH 2–12. To each of the conical flasks, 0.1 g of KMC or KMCM was contacted and allowed to stand for 48 h. The final pH of each mixture was determined and a plot of the final pH versus the initial pH was obtained from which the pH_PZC_ of KMC and KMCM was deduced from the line intercept.

## Results and discussion

**Figure 1:**
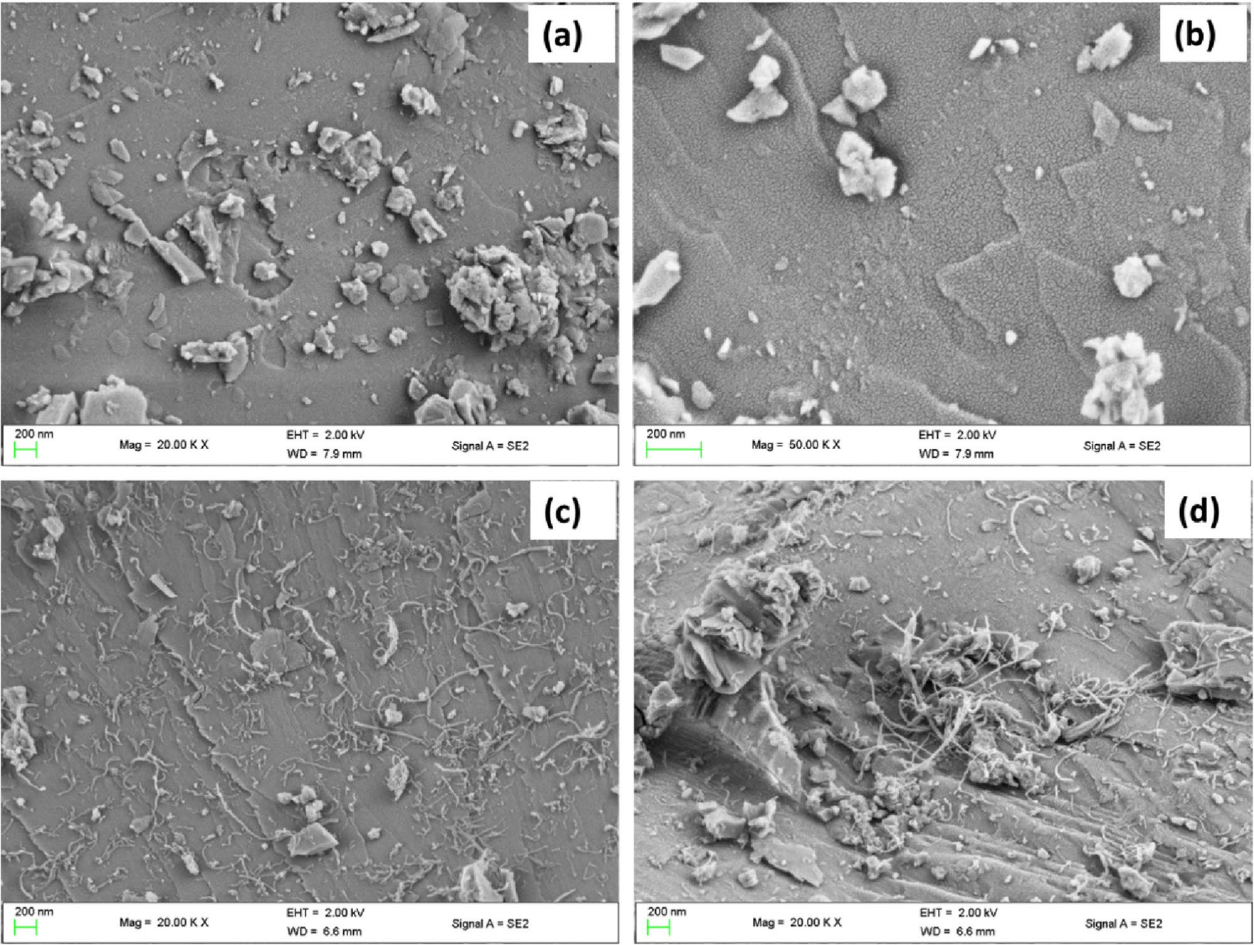
FESEM image of (**a**) KMC, (**b**) KMC-Tatz, (**c**) KMCM and (**d**) KMCM-Tatz.

The surface morphology of KMC, KMC-Tatz, KMCM and KMCM-Tatz were visualized using FESEM spectroscopic technique. The result of FESEM analysis was displayed in Fig. 1. The external micro-graphical structure of the KMC, showed clusters of irregularly shaped particles embedded on a sheetlike surface having a homogenous texture (see Fig. 1). Meanwhile, after the adsorption of Tatz onto KMC, the KMC-Tatz micrograph was noticed to have a smooth surface with ultrathin fragments of sheets in layers, suggesting that the surface of KMC was covered with Tatz (see Fig. 1). On the other hand, the micrograph acquired for KMCM and KMCM-Tatz exhibited heterogonous characteristics consisting of a weblike long cylindrical structure on sheets of irregular shapes. However, KMCM-Tatz tends to sustain a non-porous and smooth surface, indicating the coverage of KMCM by tartrazine molecules.

**Figure 2:**
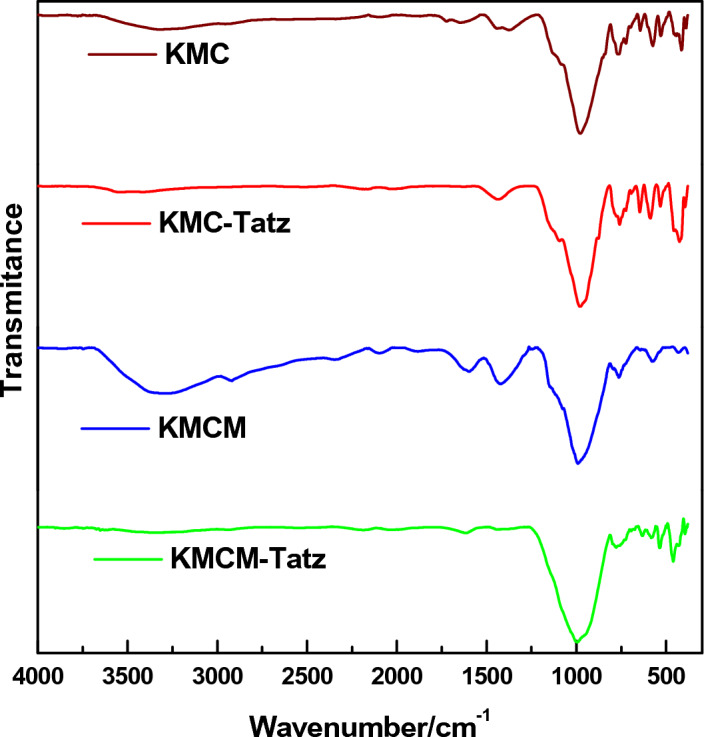
The FTIR spectra of KMC, KMC-Tatz, KMCM and KMCM-Tatz.

The functional groups on the surface of KMC and KMCM were investigated by making use of the FTIR spectrophotometer. Figure 2, showed the compared spectra of the Tatz-loaded and unloaded adsorbents. Characteristic peaks of microcline were observed at 467 cm^−1^, 586 cm^−1^, 640 cm^−1^, and 1003 cm^−1^ and were attributed to –Si–O asymmetrical bending, O–Si–(Al)–O symmetrical bending, Al–O co-ordination, and Si–(Al)–O stretching vibrations^65^. The intensity of the KMC peaks was noticed to reduce on the FTIR spectra acquired for the nanocomposite (KMCM). This could be attributed to the masking effect of the modifier (*f-*MWCNTs). Meanwhile, after the modification step, a broad peak at 3425 cm^−1^ was observed for the pristine nanocomposite (KMCM) and this was attributed to the vibration of –O–H and –NH bonds arising from the functionalization of the MWCNT^57^. On the other hand, the FTIR spectra acquired for spent KMC (KMC-Tatz) and KMCM (KMCM-Tatz) were noticed to have diminished –OH bands as well as a shift in the bands of their peaks. This could be associated with the fixation of the Tatz molecules on the surface of KMC and KMCM. The outcome of this analysis further justifies the observation made from the FESEM micrograph of the spent adsorbents.

### XRD characterization

**Figure 3:**
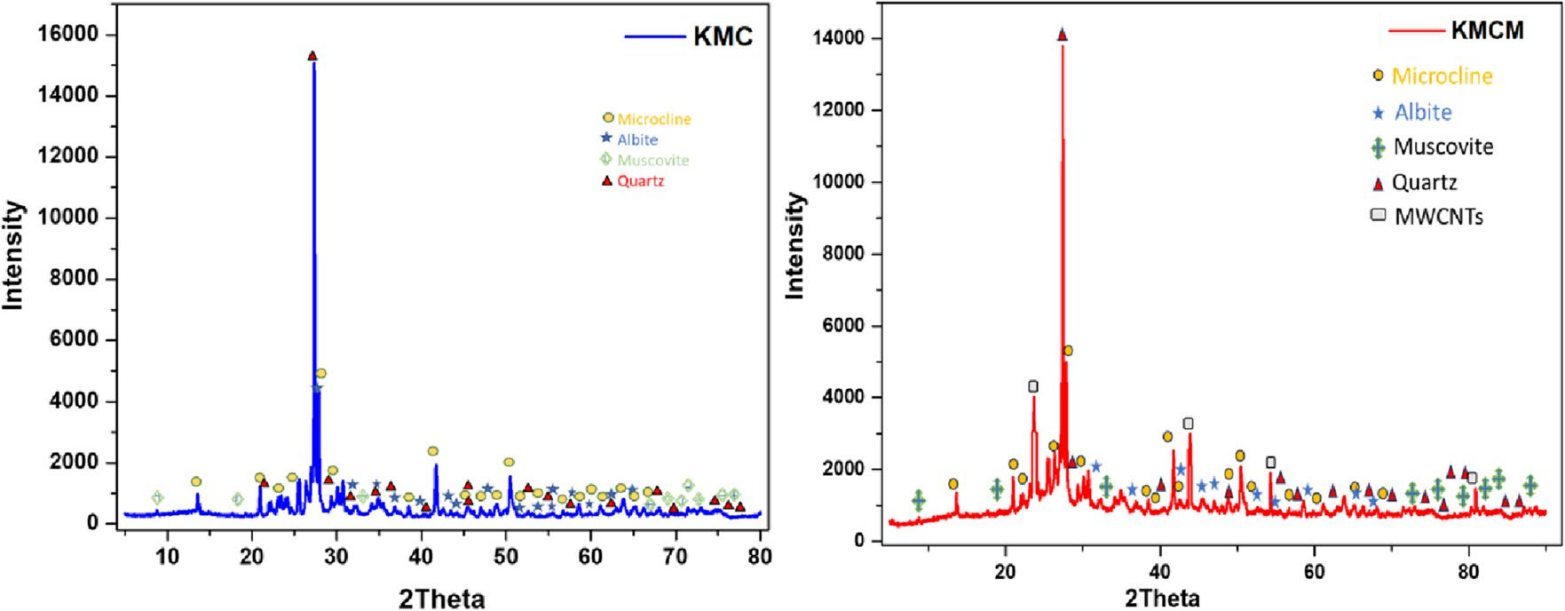
The XRD diffractograms of KMC and KMCM.

The structure of KMC was identified by making use of X-ray diffraction spectra acquired within the 2theta range of 5.0–80° (see Fig. 3). The spectra revealed that the mineral (KMC) contained a varied percentage of quartz, muscovite, albite, and microcline. The diffraction peaks of KMC are in good agreement with the reference library ICDD 19-926. Meanwhile, the XRD diffractogram of the microcline used for the adsorption of methylene blue showed similar identical diffraction peaks with our report^66^. On the other hand, the X-ray diffraction spectra obtained for KMCM showed no significant change with respect to the 2Theta values when compared to KMC. However, the reduced intensity of the peaks and formation of new peaks at 2Theta 23.43, 43.24 and 54.07 was observed and were attributed to the reflections of graphite from MWCNTs respectively (ICDD No. 01-074-2379). This reflects the homogeneity and the consistency in the crystallinity of the nanocomposite.

### Thermal analysis

**Figure 4:**
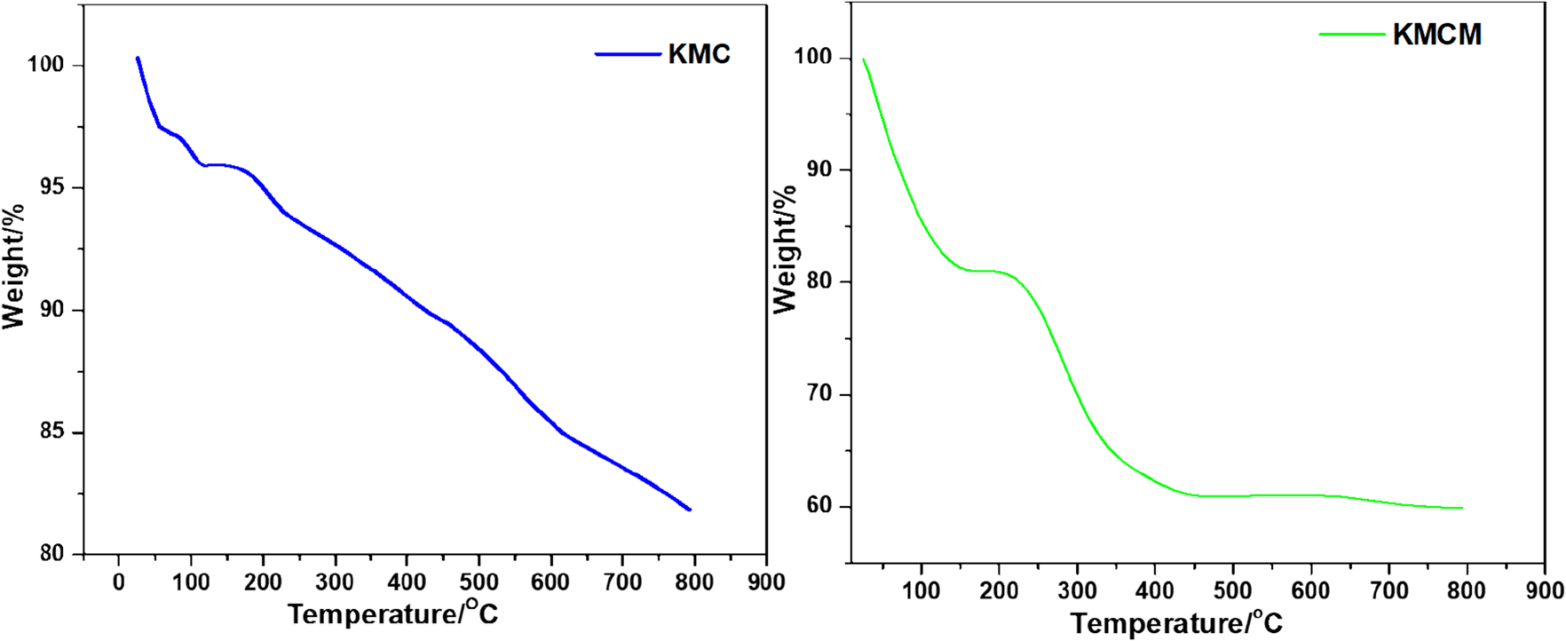
The TGA thermograms of KMC and KMCM.

The thermal stability and behaviour of KMC and KMCM were validated by making use of the thermo-gravimetric analysis (TGA). Weight loss below 200 °C is attributed to the loss of hygroscopic water from the constituent of KMC and KMCM (see Fig. 4). Meanwhile, the thermogram of KMC revealed the consistent loss of mass from 200 to 800 °C, this could be associated with the loss of constitutional water resulting from the evolution of hydroxides within the quartz, albite, and muscovite network in KMC^67^. Within the investigated temperature range, KMCM was noticed to have a three-stage degradation phase. This could be attributed to surface-bound water, volatile inorganic constituents of the nanocomposite and amorphous carbon (MWCNTs). It was observed that about 17.39 wt% (KMC) and 39.74 wt% (KMCM) were degraded over the range of temperature (25–800 °C) employed (see Fig. 4). This shows the implication of the modification step and the robust thermal stability of KMCM.

**Figure 5:**
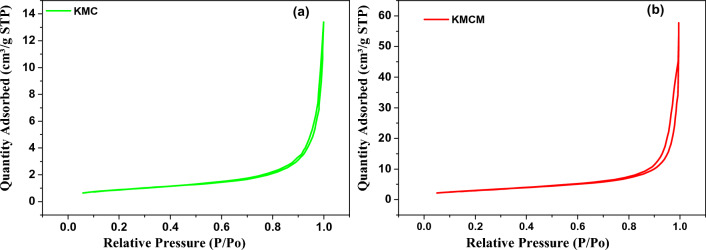
The nitrogen adsorption–desorption curves of (**a**) KMC and (**b**) KMCM.

The specific surface areas and pore size distribution of the adsorbents (KMC and KMCM) were evaluated from the N_2_ sorption–desorption isotherms techniques at 77 K. From Fig. 5, it can be observed that the specific surface area of KMCM (10.77 m^2^/g) was larger than that of KMC (3.223 m^2^/g) (see Table 1). Meanwhile, a similar result was observed from the pore size and pore volume estimation made by making use of the Barrett–Joyner–Halenda (BJH) approach. It shows that the modification of the microcline rock (KMC) with *f-*MWCNTs enhanced the surface area of the nanocomposite, which further justifies the higher uptake of the composites (KMCM) due to an increase in the available active sites (see Fig. 5) The isotherm acquired from the adsorption–desorption of N_2_ by the adsorbents were observed to correspond to type II with H3 hysteresis loop of the IUPAC classification.

**Table 1:** Textural properties of adsorbents.

Adsorbents	Surface area/m^2^ g^−1^	Pore volume/cm^3^ g^−1^	Pore diameter/nm	pH_PZC_
KMC	3.223	0.016	21.96	5.6
KMCM	10.77	0.076	27.00	4.3

### Effect of pH

**Figure 6:**
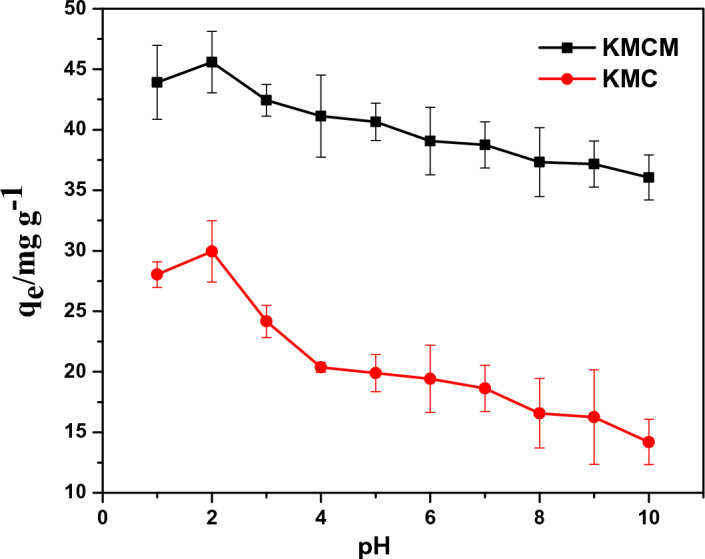
The effect of solution pH on the removal capacity of KMC and KMCM [conditions: 25 cm^3^ of 100 mg dm^−3^ Tatz, 24 h contact time, 0.05 g of dosage, agitation speed 120 rpm, temperature 25 °C].

**Figure 7:**
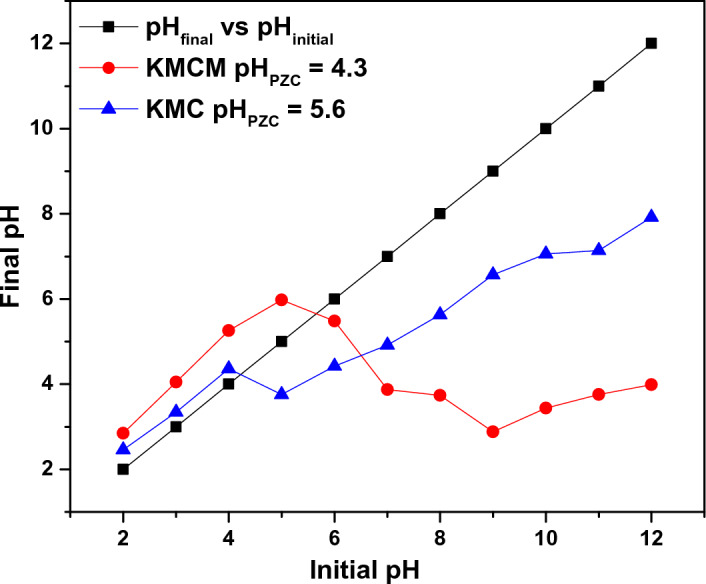
pH_PZC_ plots of KMC and KMCM.

The pH effect on Tatz adsorption onto KMC and KMCM was assessed in the range of pH 1.0 to 10.0 and the results obtained are displayed in Fig. 6. When the pH values increased from 1.0 to 2.0, the uptake capacity of KMC and KMCM increased to 29.72 mg g^−1^ and 45.86 mg g^−1^ respectively. Above these values, the removal potential of KMC and KMCM decreased. The poor Tatz removal by KMC and KMCM at solution pH 1 and pH > 2 may be attributed to the partial dissolution of the adsorbent surface and the competition with increasing OH^−^ ions as solution pH increases respectively. This phenomenon can be explained by making use of the pH_ZPC_ of KMC and KMCM. The pH_PZC_ of KMC and KMCM were determined as 5.6 and 4.3 respectively (see Fig. 7). This shows that at pH higher and lower than these values (5.6 (KMC) and 4.3 (KMCM)), the surface of the KMC and KMCM will be negatively and positively charged respectively. This suggests that at pH 2, Tatz may exist in their monomeric forms and this could enhance, easy pore capture of the Tatz molecules, but as the solution pH increases beyond pH 2, the -OH groups increases and may repel the sticking of the Tatz to the surface of KMC and KMCM. Hence, hydrophobic interaction and Tatz entrapment in the pores of the nanocomposites may be the mechanism responsible for Tatz adsorption onto the surface of KMC and KMCM. Our results are in good agreement with the report of Tatz adsorption onto other adsorbents^30^.

### Kinetics study

**Figure 8:**
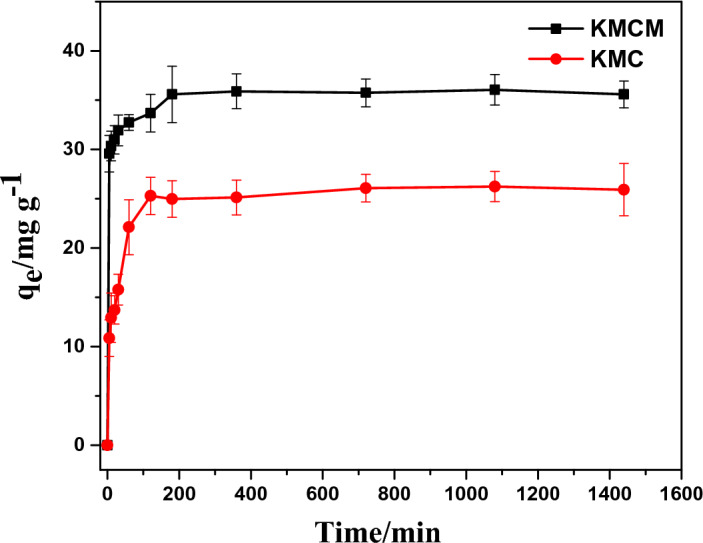
Effect of agitation time on the removal of Tatz by KMC and KMCM [conditions: 25 cm^3^ of 100 mg dm^−3^ Tatz, solution pH of 2, 0.05 g of dosage, agitation speed 120 rpm, temperature 25 °C].

**Figure 9:**
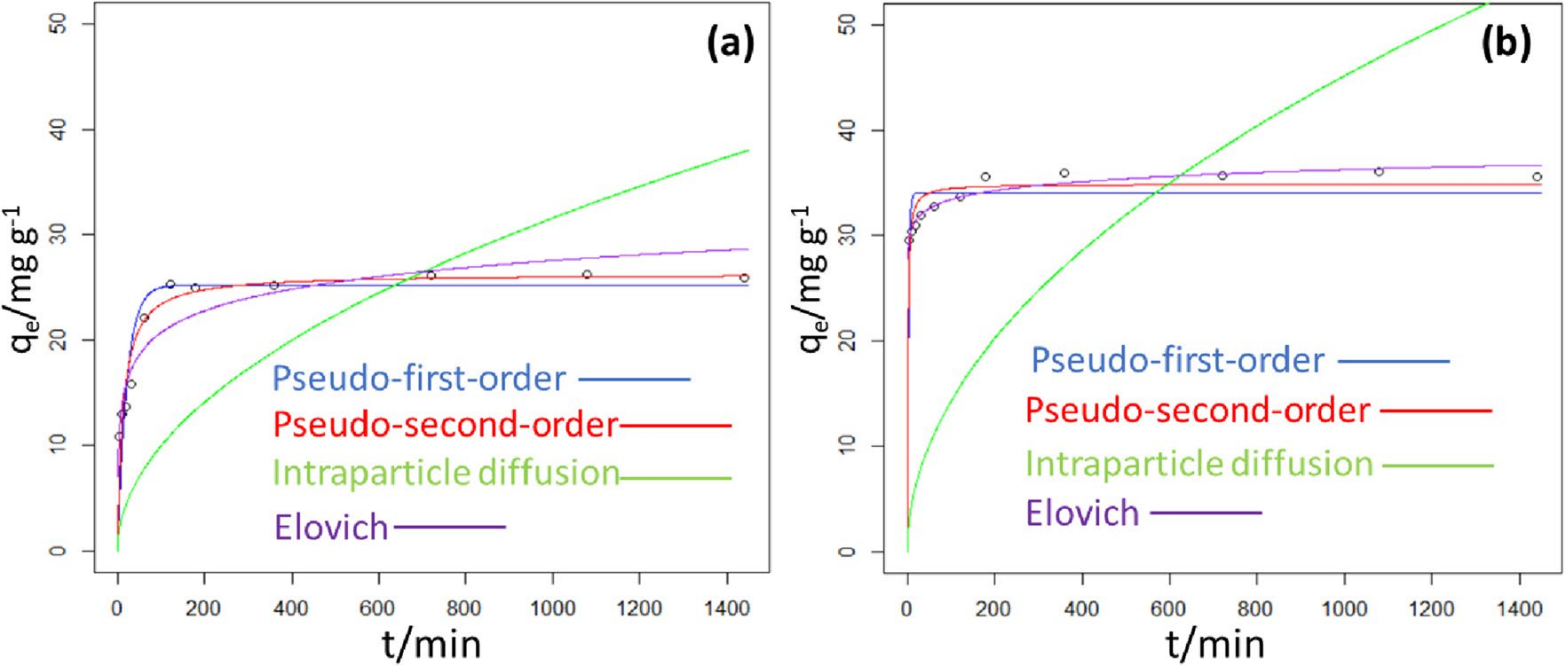
Experimental results of Tatz adsorption onto KMC and KMCM: a comparison of several kinetic models (pseudo-first order, blue line; pseudo-second-order, red line; intraparticle diffusion, green line; Elovich, purple line).

The implication of agitation time on the adsorption of Tatz was investigated by varying the agitation time from 5 to 1440 min. The outcome of this study showed that the uptake of Tatz by KMC and KMCM was in two phases (see Fig. 8). These phases include the fast and the slow phase, similar behaviour has been reported for Tatz removal by surfactant-ionic liquid bi-functionalization of chitosan beads^68^ and zinc-aluminum layered double hydroxide^30^. The elimination of Tatz by KMC and KMCM were optimum at 120 and 180 min respectively, above these optimum contact times, an insignificant amount of the Tatz was removed. However, to ensure equilibrium attainment, 1440 min was used for further experiments. The fast phase may be due to the availability of sufficient active sites on the surfaces of KMC and KMCM at the early stage of the adsorption. Meanwhile, the slow phase may probably be due to insufficient active sites on the surface of the adsorbents or as a result of pore entrapment of the Tatz molecules. To better understand the mechanism responsible for the removal of Tatz from the simulated wastewater, experimental data acquired from the time-dependent uptake study for Tatz removal by KMC and KMCM were fitted into four mathematical empirical kinetics models namely pseudo-first-order, pseudo-second-order, Elovich and Weber-Morris intraparticle diffusion via the non-linear least square analysis (nlls) (see Fig. 9). The least sum of squared residuals (SSR) and residual square errors (RSE) of the different kinetic models were compared and the model with the least values was selected as the model that best describes the mechanism accountable for the elimination of Tatz over the studied duration. As displayed in Table 2, the uptake of Tatz by KMC was best expressed by pseudo-second-order kinetics. On the other hand, Elovich kinetic model was observed to best describe the kinetics involved in the removal of Tatz by KMCM. Pseudo-second-order kinetics suggest that the elimination of Tatz by KMC is a chemisorption process involving a bimolecular sorbate-sorbent adsorptive interaction. Our results corroborate with the uptake of Tatz onto Katira gum-*cl*-*poly*(acrylic acid-co-*N*-vinyl imidazole) hydrogel^69^ and polyamidoamine dendrimer gel^70^.

**Table 2:** Kinetics parameters for Tatz removal onto KMC and KMCM for the indicated kinetic models.

Model	Parameter	KMC	KMCM
Experimental	q_exp_/mg g^−1^	26.06	35.99
Pseudo first order	K_1_/min^−1^	0.047	0.368
	q_eq_/mg g^−1^	25.17	34.03
	SSR	61.63	39.47
	RSE	2.617	2.094
Pseudo second order	K_2_/g mg^−1^ min^−1^	0.0031	0.0231
	q_eq_/mg g^−1^	26.29	34.89
	SSR	29.24	16.77
	RSE	1.803	1.365
	h	2.116	28.168
	t_0.5_/min	12.422	1.2365
Intraparticle diffusion	K_id_/mg g^−1^ min^−0.5^	0.9984	1.429
	l/mg g^−1^		
	SSR	1134	4163
	RSE	10.65	20.4
Elovich	α/mg g^−1^ min^−1^	7.045	27.76
	β/g mg^−1^	2.965	1.229
	SSR	49.99	4.528
	RES	2.357	0.709

Meanwhile, the adsorption of Tatz by KMCM is predominantly a chemisorption process. This suggests that the elimination of Tatz by both sorbents involves sharing or exchange of electrons. The mechanistic kinetics model (intraparticle diffusion concept) was used to assess the possible steps involved in the transfer of Tart molecules from the bulk solution into pores of KMC and KMCM as well as external surface adsorption. The intraparticle diffusion rate constant for the uptake of Tatz onto KMC and KMCM was estimated from the plot of the square root of time (t^1/2^) against the uptake capacity of the KMC and KMCM. From the plot, a linear relationship with the line passing through the origin of the graph indicates that the rate-determining step is intraparticle diffusion. On the contrary, non-zero intercepts were extrapolated from the plots, and this indicates a difference in mass transfer rate between the early and late stages of the adsorption process. It also indicates that intraparticle diffusion may not be the sole rate-determining step in the adsorption of Tatz onto KMC and KMCM.

### Effect of adsorbents dosage

**Figure 10:**
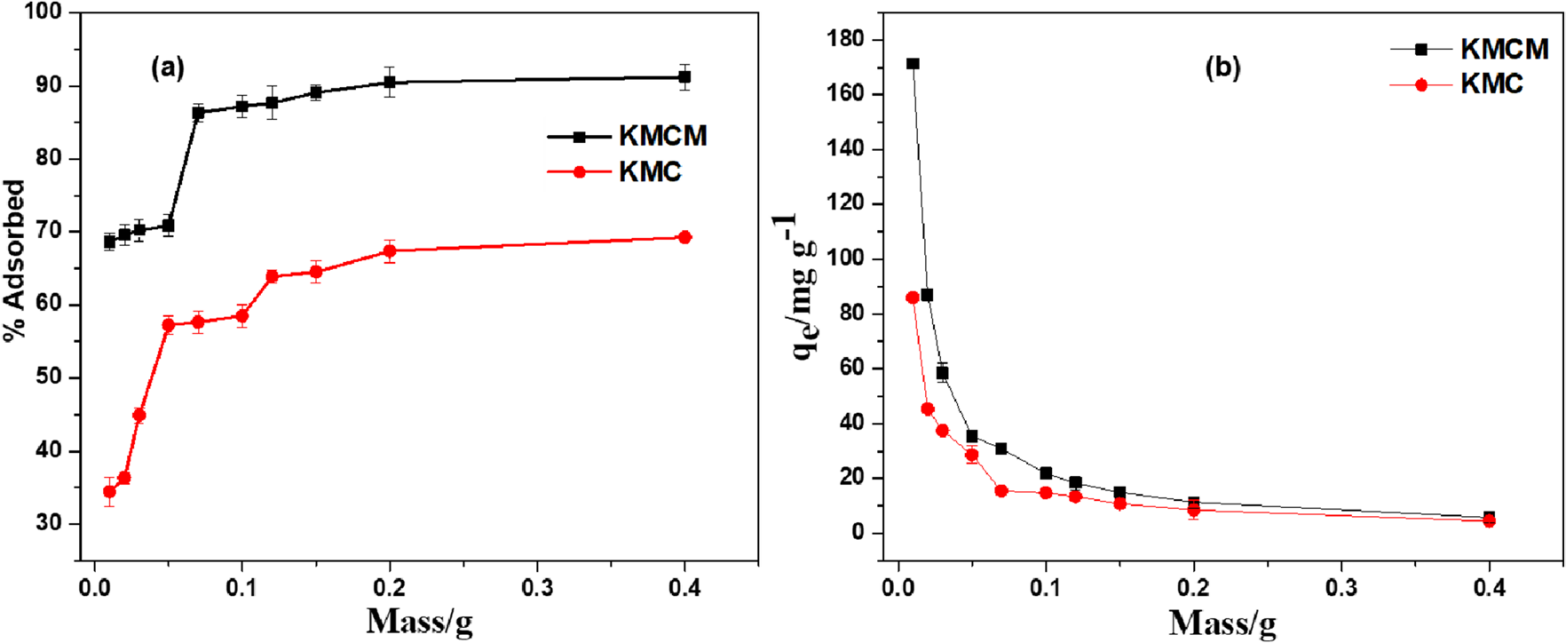
Effect of dosage on the uptake capacity (q_eq_) of Tatz adsorption by KMC and KMCM. [conditions: 25 cm^3^ of 100 mg dm^−3^ Tatz, solution pH of 2, 24 h contact time, agitation speed 120 rpm, temperature 25 °C].

By changing the sorbent mass from 0.01 to 0.4 g, the effect of the adsorbent dose on the removal of Tatz from the aqueous was examined. An increase in the uptake efficiency of KMC and KMCM was observed with an increase in the adsorbent dose (see Fig. 10). This could be attributed to the increasing number of chemical moieties per adsorbent unit with the increase in dosage^71^. The sorbate-to-sorbent ratio was noticed to decrease with an increase in the dosage of KMC and KMCM (see Fig. 10). This phenomenon could be due to the disproportional increase of the active sites on the sorbent surface and sorbent mass^72^, it could also be due to an increase in the active sorbent mass with fixed sorbate concentration. At the end of this study, 0.05 g was selected as the optimum mass for both adsorbents and was used for further study. Above this mass, insignificant uptake of Tatz was observed.

### Effect of solution temperature and initial Tatz concentration

**Figure 11:**
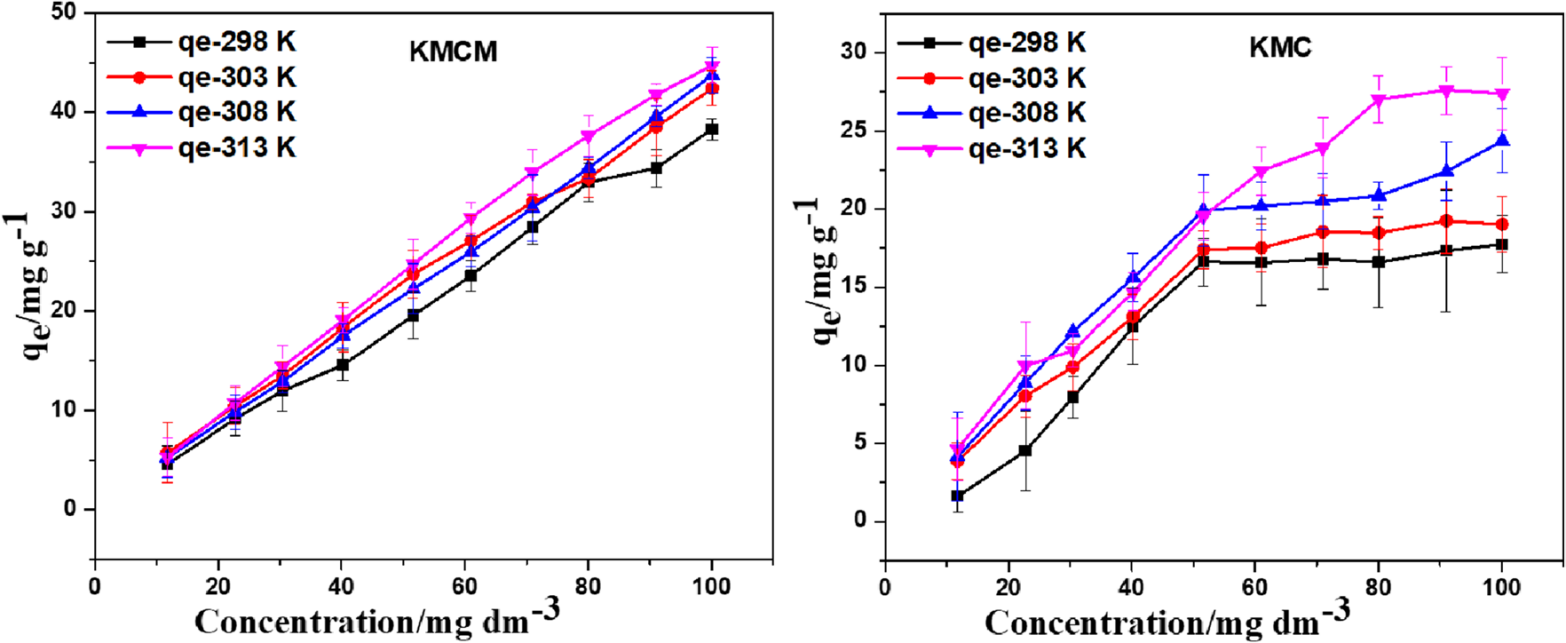
The implication of starting Tatz concentration and temperature on the adsorption potential of KMC and KMCM for Tatz [conditions: 25 cm^3^ of Tatz solution, pH of 2, 24 h contact time, agitation speed 120 rpm, 0.05 g of adsorbent dose, temperature of 25 °C].

As displayed in Fig. 11, increased uptake capacity of KMC and KMCM for Tatz were observed with an increase in the initial Tatz concentration. This could be due to the decrease of the resistance to mass transfer with increased Tatz concentration. At high concentrations of Tatz, the Tatz molecules are readily available at the binding sites and pores of KMC and KMCM. The adsorption potential of the unused adsorbents KMC was noticed to increase from 10 to 50 mg dm^−3^ above which a gradual increase is observed. Meanwhile, the nanocomposite showed a consistent increase in its uptake capacity with increased Tatz concentration. This suggests that the surface of KMCM consists of sufficient active sites that are capable of eliminating high Tatz concentrations in the aquatic ecosystem. To investigate the implication of solution temperature on the elimination of Tatz from the aquatic ecosystem, the effect of the initial concentration of Tatz experiment was repeated at different temperatures (298, 303, 308 and 313 K). The uptake capacity of the adsorbents was slightly enhanced with an increase in solution temperature, this suggests that the performance of the adsorbents was temperature dependent, also these adsorbents can be effective at elevated solution temperature.

The equilibrium process involved in the uptake of the Tatz molecule from the aqueous phase can be used to investigate the pattern of adsorbent-adsorbate interactions. Meanwhile, in the design of a large-scale adsorption process, an understanding of the equilibrium process guides the optimization of the adsorbent^73^. The Redlich-Peterson models, Freundlich, Sips, Temkin, Dubinin Radushevick, Khan, Toht, and Langmuir isotherm analysis were all used in this work.

Data from experiments on the impact of the starting concentration and solution temperature experiment was fitted into the aforementioned isotherm models via the nls nonlinear regression routine. Models with the least SSR and RSE values were assumed to have the capacity to best describe the experimental data. Among the two-parameter models, the Langmuir adsorption isotherm was observed to best explain the experimental results, this indicates a monolayer coverage of KMC and KMCM. The maximum monolayer covering capacity (*q*_*m*_) was observed to vary from 24.31 to 37.96 mg g^−1^ and 37.66 to 67.17 mg g^−1^ for KMC and KMCM respectively. A comparison of the monolayer adsorption capacity acquired for KMC and KMCM with other adsorbents showed the superior nature of KMCM (see Table 3). Meanwhile, the Langmuir adsorption constant (*b*), of KMCM was noticed to be higher than KMC (see Tables 4 and 5). This shows that the modification of this mineral (microcline) with *f-*MWCNTs did enhance the adsorptive force of the nanocomposite with increased solution temperature, hence, an increase in the sorbate to sorbent ratio of the adsorbents was observed. Amongst the three-parameter isotherms, the Sips model was noticed to best fit the experimental data. The Sips model also revealed the little contribution of heterogenous coverage from the non-unit *n* values estimated over the range of temperature investigated (see Tables 4 and 5).

**Table 3:** Comparison of the Langmuir maximum adsorption capacities of Tatz for KMC and KMCM with those of other sorbents.

Adsorbents	q_max_ (mg g^−1^)	References
KMC	37.96	This study
KMCM	67.17	This study
Chitin	30.00	^77^
Sawdus	4.80	^78^
Tenorite nanoparticles	42.50	^79^
Bottom ash	12.60	^80^
Hen feather	64.1	^81^
PAni/SD	2.45	^82^
Polyaniline nano layer composite	2.47	^82^
Amberlite IRA-910	50.00	^83^

**Table 4:** Isotherm parameters for the adsorption of Tatz onto KMCM.

Adsorbent	Isotherm	Parameters	295 K	303 K	310 K	318 K
KMCM	Freundlich	K_F_	7.361	8.902	2.627	14.36
		n	2.563	1.809	0.926	1.906
		SSR	330.8	60.43	234.60	326.5
		RSE	6.430	2.748	2.080	6.388
	Langmuir	q_m_	37.66	61.44	61.44	67.17
		b	0.102	0.115	0.116	0.228
		SSR	233.0	68.42	288.7	208.6
		RSE	5.397	2.924	–	5.106
	D–R	q_m_	30.15	–	–	–
		β	876.7	–	–	–
		SSR	1147	418.9	746.8	1037
	Temkin	b_T_	321.9	–	–	–
		A_T_	3.649	–	–	–
		SSR	1005	292.1	571.2	1074
	R–P	K_RP_	11.07	30.15	294.7	294.7
		α	0.017	321.9	0.030	0.030
		β	1.950	1.906	1.650	1.650
		SSR	138.8	1846	60.22	272.8
		RSE	4.453	–	2.933	–
	Sip	q_m_	30.15	294.7	294.7	294.7
		b	321.9	0.030	0.030	0.030
		n	1.906	1.650	1.650	1.650
		SSR	124	60.22	172.8	133
		RSE	–	2.933	–	–
	Toth	q_m_	294.7	294.7	294.7	294.7
		K_T_	11.07	11.07	11.07	11.07
		n_T_	1.650	1.650	1.650	1.650
		SSR	59,516	52,233	61,831	44,529
	Khan	q_m_	294.7	294.7	294.7	294.7
		b_K_	0.030	0.030	0.030	0.030
		a_K_	0.017	0.017	0.017	0.017
		SSR	259,133	27,266	23,526	3289
		RSE	–	–	–	–

**Table 5:** Isotherm parameters for the adsorption of Tatz onto KMC.

Adsorbent	Isotherm	Parameters	295 K	303 K	310 K	318 K
KMC	Freundlich	K_F_	2.494	4.563	6.247	5.448
		n	2.013	2.673	2.866	2.200
		SSR	120.4	49.69	74.08	66.55
		RSE	3.880	2.492	3.043	2.884
	Langmuir	q_m_/mg g^−1^	26.89	24.31	27.31	37.96
		b/dm^3^ mg^−1^	0.038	0.082	0.121	0.073
		SSR	98.61	21.72	38.95	44.58
		RSE	3.511	1.648	2.207	2.361
	D–R	q_m_	20.18	–	–	–
		β	278.7	–	–	–
		SSR	409.9	42.13	114.3	377.2
	Temkin	b_T_	449.8	–	–	–
		A_T_	0.937	–	–	–
		SSR	291.4	58.98	72.31	268.6
	R–P	K_RP_	–	1.258	1.259	1.903
		α	–	0.005	0.005	0.007
		β	–	1.564	1.564	1.497
		SSR	268.6	188.8	188.8	40.70
		RSE	–	–		2.411
	Sip	q_m_	20.18	20.18	20.18	37.52
		b	449.7	149.8	49.8	0.072
		n	2.199	2.199	2.199	0.983
		SSR	60.4	59.5	191.4	44.57
		RSE	–	–	–	2.523
	Toth	q_m_	42.71	60.46	60.46	130.2
		K_T_	156.83	68.73	68.73	165.6
		n_T_	1.113	1.270	1.270	1.403
		SSR	197.89	180.26	180.6	139.96
		RSE	3.740	1.615	–	–
	Khan	q_m_	130.2	140.9	140.9	140.9
		b_K_	718.6	0.010	0.010	0.010
		a_K_	0.007	3.194	3.194	3.194
		SSR	1,009,824	11.80	184.2	441.2
		RSE	–	1.298	–	–

### Thermodynamic parameters of adsorption

Entropy change (∆S°), enthalpy change (∆H°) and free energy change (∆G°) are cardinal thermodynamic parameters that depend on the temperature of the adsorption process^74^. These parameters give insight into the implication of temperature on the sorbent-sorbate interaction, the path of heat flow during the adsorption process and the nature of binding forces responsible for sorbate removal. These parameters were estimated by making use of Van’t Hoff equations^75^.

1 $$\Delta G^\circ =-RT\mathrm{ln}{K}_{L}$$

2 $$\mathrm{ln}{K}_{L}=-\frac{\Delta {H}^{^\circ }}{RT}+\frac{\Delta S^\circ }{R}$$

T is the temperature in Kelvin, K_L_ is the corrected dimensionless constant calculated from the product of the Langmuir constants q_m_, 1000 and b in dm^3^ mol^−1^^76^, and R is the universal gas constant (8.314 J mol^−1^ K^−1^). At all temperatures studied, the ΔG° were negative (see Table 6), this suggests that the removal process is spontaneous and feasible. Meanwhile, the ΔG° values were more negative with increased solution temperature, this indicates that the reaction is favoured at high solution temperature. In addition to the implication of solution temperature, the estimated ∆H° and ∆S° values were positive, this implies that the removal process of Tatz onto KMCM and KMC is endothermic and entropy-driven respectively. Hence, KMCM has demonstrated the potential to eradicate dyes from the aqueous solution and can be scaled up for environmental remediation practices.

**Table 6:** Thermodynamic factors for adsorption of Tatz onto KMCM and KMC.

Adsorbents	T/K	ΔG°/kJ mol^−1^	ΔH/ kJ mol^−1^	ΔS/J K^−1^ mol^−1^
KMCM	298	− 20.2452	42.91	214.2
	303	− 22.3396		
	310	− 22.8557		
	318	− 25.476		
KMC	298	− 16.9788		
	303	− 19.1281	35.01	177.6
	310	− 20.8879		
	318	− 20.9625		

### Adsorption mechanism

**Figure 12:**
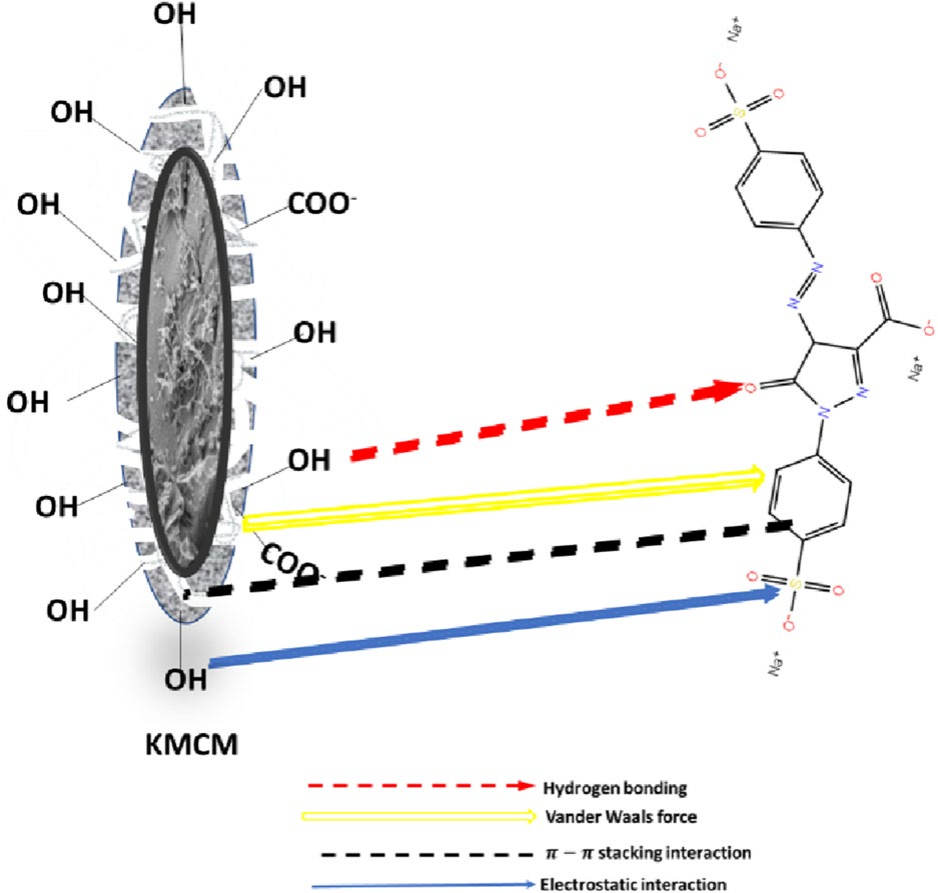
Schematic representation of the mechanism of Tatz adsorption onto KMCM.

The choice of adsorbents in water decontamination via the batch adsorption technique is a function of the adsorbate chemistry as well as the surface characteristics of the adsorbent. The aforementioned determines the nature of sorbate-sorbent interaction. To verify the adsorptive mechanism responsible for the sequestration of tartrazine by KMCM, spectroscopic techniques (SEM and FTIR) and factors of adsorption experiments (solution pH and contact time) were employed. Deduction from the SEM analysis revealed the fixation of Tatz to the surface of KMCM. Meanwhile, FTIR spectra acquired for Tatz-KMCM showed decreased peak intensity and shift in adsorption bands. This indicates the possibility of chemisorption, which was further justified using the kinetic model assessment. Elovich kinetic model was noticed to describe the uptake of Tatz onto KMCM best. On the other hand, optimum adsorption by KMCM was achieved in the acidic medium (pH = 2). Hence, the Na atoms of tartrazine dye may be substituted by proton to form 3-carboxy-5-hydroxy-1- p-sulfophenyl-4-psulfophenylazopyrazole and can also protonate the nitrogen atoms of tartrazine. In summary, we propose that these charged sites on the adsorbate may bind with the hydroxyl functional groups on the surface of KMCM via electrostatic interaction (see Fig. 12). Owing to the hydrophobic nature of the modifier (MWCNTs) that was used for the adsorbent fabrication and the overall functional group on the adsorbent surface, the contribution of Van der Waals force hydrogen bonding, π–π stacking interaction in the removal of Tatz is certain^84,85^.

### Reusability

**Figure 13:**
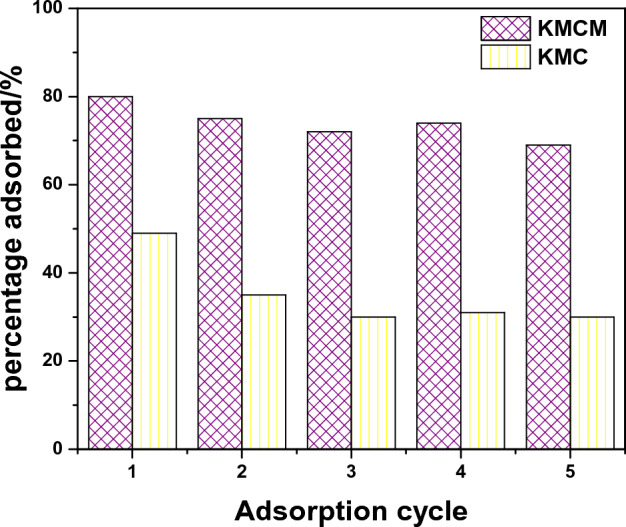
The Tatz adsorption efficiency of KMC and KMCM after different cycles.

The recyclability of an adsorbent gives insight into the economic benefit of the material. The reusability of KMC and KMCM was investigated by performing adsorption followed by desorption. The reusability of KMC and KMCM for Tatz uptake was examined using NaOH as an eluting agent. As demonstrated in Fig. 13, after the fifth cycle, KMC and KMCM exhibited efficiency levels of approximately 30% and 71%, respectively. The reduced efficiency with increased usage may be attributed to the loss of binding sites. Hence, the nanocomposite has shown the capacity for industrial wastewater treatment practice.

## Conclusion

In summary, we have successfully synthesized and characterized microcline-based nanocomposite for improved adsorption of tartrazine. FTIR analyses confirm the modification of microcline using MWCNTs and the incorporation of Tatz to the surface of KMC and KMCM. The uptake of Tatz using KMC or KMCM was noticed to be strongly dependent on solution pH, initial Tatz concentration and adsorbent dose. The acidic pH is suitable for the adsorptive removal of Tatz by KMC and KMCM. Meanwhile, optimum adsorptive conditions such as pH 2, 0.05 g adsorbent dose, 180 min contact time and 100 mg dm^−3^ initial concentration were established. The Langmuir isotherm model was found to best reflect the elimination of Tatz from KMC and KMCM. Meanwhile, the maximal monolayer uptake capacities of KMC and KMCM were determined to be 37.96 mg g^−1^ and 67.17 mg g^−1^, respectively. The uptake kinetics shows that the pseudo-second-order and Elovich models express well the adsorption of Tatz onto KMC or KMCM respectively. Hence, it is inferred that KMC and KMCM were found to be promising for the effective removal of Tatz from the aquatic ecosystem.

## Supplementary Information

Supplementary Information.

## Data Availability

The authors declare that all data generated and analysed are available within the manuscript [and its supplementary information file].
