# Supplementary material for: Thermodynamics, kinetics and isothermal studies of tartrazine adsorption onto microcline/MWCNTs nanocomposite and the regeneration potentials
Source: Sci Rep. 2023 Jun 19;13:9872. doi: 10.1038/s41598-023-37181-2 (PMC10279646; doi:10.1038/s41598-023-37181-2)
Supplement: Supplementary file 1 — Supplementary Information. [file 41598_2023_37181_MOESM1_ESM.docx]

**Thermodynamics, kinetics and isothermal studies of tartrazine adsorption onto microcline/MWCNTs nanocomposite and the regeneration potentials**

**James Friday Amaku and Raymond Taziwa**

Department of Applied Science, Faculty of Science Engineering and Technology, Walter Sisulu University, Old King William Town Road, Potsdam Site, East London 5200, South Africa.

* Correspondence: famaku@wsu.ac.za

**SUPPLEMENTARY INFORMATION**

**Calculation of percentage removal and uptake capacity**

The adsorption capacity and adsorption efficiency of the adsorbents were calculated by making use of Equation (S1), and (S2) respectively:

$q_{eq}=\left( \frac{C_{i}-C_{eq}}{m} \right)V$ (S1)

$\% adsorbed=\left( \frac{C_{i}-C_{eq}}{C_{i}} \right)\times100$ (S2)

where C_i_ is the initial Tatz concentration (mg dm^-3^), C_eq_ is the equilibrium concentration of Tatz (mg dm^-3^), m is the adsorbent mass (g), and V is the volume of Tatz solution (dm^3^).

**Kinetics and isotherm models**

Experimental data obtained from the kinetic experiment were using the pseudo-first order, pseudo-second order, intraparticle diffusion, and Elovich kinetics models as shown in Table S1. On the other hand, the experimental data obtained from initial Tatz concentration experiment over a known temperature range (298-313 K) were fixed into eight isotherm models which include Freundlich, Langmuir, Temkin, Dubinin–Radushkevick, Sips, Khan, Redlich–Peterson, Toth isotherm models as shown in Table. S2.

**Table S1** Kinetics models investigated for the adsorption of Tatz onto KMC and KMCM.

| Kinetic models | Equations | Parameters | References |
| --- | --- | --- | --- |
| Pseudo-first order | $\frac{{dq}_{t}}{d_{t}}=k_{1}\left( q_{e}-q_{t} \right)$ | $q_{e}{,k}_{1}$ | [1] |
| Pseudo-second order | $\frac{{dq}_{t}}{d_{t}}=k_{2}\left( q_{e}-q_{t} \right)^{2}$ | $k_{2},q_{e}$ | [2] |
| Weber-Morris intraparticle diffusion | $\frac{{dq}_{t}}{d_{t^{-0.5}}}=k_{id}$ | $k_{id,} l$ | [3] |
| Elovich | $\frac{{dq}_{t}}{d_{t}}=\alpha exp\left( -\beta q_{t} \right)$ | $\alpha, \beta$ | [4] |
| k_1_, pseudo-first order rate constant (min^-1^); k_2_, pseudo-second order rate constant (g mg^-1^ min^-1^). k_id_, intraparticle diffusion rate constant (mg g^-1^ min^0.5^); l, is a constant related to the boundary layer thickness (mg g^-1^);q_t_, quantity of adsorbate adsorbed at time t (mg g^-1^); q_e_, quantity of adsorbate adsorbed at equilibrium (mg g^-1^); α, adsorption rate constant (mg g^-1^ min^-1^); β, desorption rate constant (g mg^-1^). | | | |

**Table S2** Isotherm models used to describe the uptake of Tatz onto KMC and KMCM.

| Isotherm model | Equation | Parameters | References |
| --- | --- | --- | --- |
| Langmuir | $q_{e}=\frac{q_{max}{bC}_{e}}{1+bC_{e}}$ | $q_{max},$b | [5] |
| Freundlich | $q_{e}=K_{F}C_{e}^{\frac{1}{n}}$ | $k_{F}, n$ | [6] |
| Temkin | $q_{e}=\frac{RT}{b_{T}}\ln\left( A_{T}C_{e} \right)$ | $A_{T}{, b}_{T}$ | [7] |
| Dubinin-Radushkevich | $q_{e}=q_{max}e^{{-\beta\varepsilon}^{2}}$  $\varepsilon=RT\ln\left( 1+\frac{1}{C_{e}} \right)$ | $q_{max}, \beta$ | [8] |
| Sips | $q_{e}=\frac{{bq}_{max}C_{e}^{\frac{1}{n}}}{1+{bC}_{E}^{\frac{1}{n}}}$ | $q_{max}, b, n$ | [9] |
| Toth | $q_{e}=\frac{q_{max}C_{e}}{\left( \frac{1}{K_{T}}+C_{e}^{n_{T}} \right)^{\frac{1}{n_{T}}}}$ | $q_{max}{,K}_{T},n_{T}$ | [10] |
| Redlich-Peterson | $q_{e}=\frac{K_{RP}C_{e}}{1+a_{RP}C_{e}^{g}}$ | $K_{RP}{, a}_{RP,} g$ | [11] |
| Khan | $q_{e}=\frac{q_{max}b_{K}C_{e}}{\left( 1+b_{k}C_{e} \right)^{a_{k}}}$ | $q_{max,}a_{K,}b_{K}$ | [12] |

q_eq_, adsorption capacity (mg g^-1^); C_eq_, equilibrium concentration of adsorbate in solution (mg dm^-3^); q_max_, maximum monolayer capacity (mg g^-1^); b, Langmuir isotherm constant (dm^3^ mg^-1^); K_F_, Freundlich isotherm constant (mg g^-1^) (dm^-3^ mg^-1^)^n^; n, adsorption intensity; b_T_, Temkin isotherm constant; A_T_, Temkin isotherm equilibrium binding constant (dm^-3^ g^-1^); b, Dubinin–Radushkevich isotherm constant (mol^2^ kJ^-2^); K_T_, Toth isotherm constant (mg g^-1^); n_T_, Toth isotherm constant; K_RP_, Redlich–Peterson isotherm constant (dm^-3^ g^-1^); a_RP_, Redlich–Peterson isotherm constant; g, Redlich–Peterson isotherm exponent; a_K_, Khan isotherm exponent; b_K_, Khan isotherm constant.

**References**

1. Aksu, Z., Karabayır, G.: Comparison of biosorption properties of different kinds of fungi for the removal of Gryfalan Black RL metal-complex dye. Bioresource Technology **99**(16), 7730-7741 (2008).

2. Sevim, A.M., Hojiyev, R., Gül, A., Çelik, M.S.: An investigation of the kinetics and thermodynamics of the adsorption of a cationic cobalt porphyrazine onto sepiolite. Dyes and Pigments **88**(1), 25-38 (2011). doi:<http://dx.doi.org/10.1016/j.dyepig.2010.04.011>

3. Ofomaja, A., Naidoo, E., Modise, S.: Removal of copper (II) from aqueous solution by pine and base modified pine cone powder as biosorbent. Journal of Hazardous Materials **168**(2), 909-917 (2009).

4. Omorogie, M.O., Babalola, J.O., Unuabonah, E.I., Gong, J.R.: Clean technology approach for the competitive binding of toxic metal ions onto MnO2 nano-bioextractant. Clean Technologies and Environmental Policy **18**(1), 171-184 (2016).

5. Langmuir, I.: The adsorption of gases on plane surface of glass, mica and platinum. Journal of the American Chemical Society **40**(9), 1361-1403 (1918). doi:10.1021/ja02242a004

6. Freundlich, H.: Over the adsorption in solution. Journal of Physical Chemistry **57**(385), e470 (1906).

7. Temkin, M., Pyzhev, V.: Kinetics of the synthesis of ammonia on promoted iron catalyst. Journal of Physical Chemistry (USSR) **13**, 851-867 (1939).

8. Dubinin, M.: The potential theory of adsorption of gases and vapors for adsorbents with energetically nonuniform surfaces. Chemical Reviews **60**(2), 235-241 (1960).

9. Sips, R.: On the structure of a catalyst surface. The Journal of Chemical Physics **16**(5), 490-495 (1948).

10. Toth, J.: State equations of the solid-gas interface layers. Acta Chimica Academiae Scientiarum Hungaricae **69**(3), 311-328 (1971).

11. Redlich, O., Peterson, D.L.: A useful adsorption isotherm. Journal of Physical Chemistry **63**(6), 1024-1024 (1959).

12. Khan, A., Al-Waheab, I., Al-Haddad, A.: A generalized equation for adsorption isotherms for multi-component organic pollutants in dilute aqueous solution. Environmental Technology **17**(1), 13-23 (1996).
